# How do accountability problems lead to maternal health inequities? A review of qualitative literature from Indian public sector

**DOI:** 10.1186/s40985-018-0081-z

**Published:** 2018-03-16

**Authors:** Mukesh Hamal, Marjolein Dieleman, Vincent De Brouwere, Tjard de Cock Buning

**Affiliations:** 1 Athena Institute for Research on Innovation and Communication in Health and Life Sciences (VU University), De Boelelaan 1085, 1081 HV Amsterdam, The Netherlands; 2 ISGlobal, Barcelona Centre for International Health Research (CRESIB), Hospital Clínic-Universitat de Barcelona, Barcelona, Spain; 3 Woman and Child Health Research Centre, Department of Public Health, Institute of Tropical Medicine, Antwerp, Belgium; 4 KIT Health, PO Box 95001, 1090 HA Amsterdam, The Netherlands

**Keywords:** Inequity, Maternal health, Health system, Accountability, India

## Abstract

**Background:**

There are several studies from different geographical settings and levels on maternal health, but none analyzes how accountability problems may contribute to the maternal health outcomes. This study aimed to analyze how accountability problems in public health system lead to maternal deaths and inequities in India.

**Methods:**

A conceptual framework was developed bringing together *accountability process* (in terms of standard setting, performance assessment, accountability (or answerability, and enforceability) —an ongoing cyclical feedback process at different levels of health system) and *determinants of maternal health* to analyze the influence of the process on the determinant leading to *maternal health outcomes*. A scoping review of qualitative and mixed-methods studies from public health sector in India was conducted. A narrative and interpretive synthesis approach was applied to analyze data.

**Results:**

An overarching influence of health system-related factors over non-health system-related factors leading to maternal deaths and inequities was observed. A potential link among such factors was identified with gaps in accountability functions at all levels of health system pertaining to policy gaps or conflicting/discriminatory policies and political commitment. A large number of gaps were also observed concerning performance or implementation of existing standards. Inherent to these issues was potentially a lack of proper monitoring and accountability functions. A critical role of power was observed influencing the accountability functions.

**Conclusion:**

The narrative and interpretive synthesis approach allowed to integrate and reframe the relevant comparable information from the limited empirical studies to identify the hot spots of systemic flaws from an accountability perspective. The framework highlighted problems in health system beyond health service delivery to wider areas such as policy or politics justifying their relevance and importance in such analysis. A crucial message from the study pertains to a need to move away from the traditional concept of viewing accountability as a blame-game approach and a concern of limited frontline health workers towards a constructive and systemic approach.

## Background

Even if maternal deaths have decreased at the global level, many women continue to die due to pregnancy- and childbirth-related causes, especially in low- and middle-income countries (LMICs). The World Health Organization (WHO) estimated that in 2015, about 99% of maternal deaths worldwide were in developing countries [1]. Direct obstetric causes account for about 73% of all maternal deaths globally, the most common being hemorrhage, hypertensive disorders, sepsis, abortions, complications of delivery, and obstructed labor [2]. The remaining deaths were due to indirect causes such as pre-existing medical conditions. Even when there are effective interventions to prevent and treat these causes, women continue to die because of the limitations of health systems and social structures preventing women from having access to health care [3].

Global strategies to achieve the Sustainable Development Goals (SDGs) have explicitly emphasized accountability in health systems and as a part of governance as a core principle to attain the SDG in relation to maternal and perinatal health [4, 5]. Although accountability has been variously defined [6, 7], the most common definition in the health sector [8–12] pertains to Schedler’s [13] two-dimensional concept: an obligation for *answerability—*to provide information about and/or justification for the actions of the bodies accountable to accounting bodies—and *enforceability*—to be the subject to some form of sanction for failure to comply with and/or engage in appropriate action by accountable bodies.

Improved accountability has been critically highlighted for better performance of health systems [7, 12, 14]. Interventions aimed at improving accountability in health systems have shown to improve health outcomes (including maternal health) in terms of availability, accessibility, and uptake of services [15, 16]. Conversely, a lack of accountability particularly in public sector has been highlighted as a major issue in LMICs where the public sector has often failed to provide adequate services to citizens [6]. Studies in maternal health have highlighted that lack of accountability as a part of governance in health service delivery (e.g., lack of grievance or redressal mechanisms, provider’s negligence during delivery, irrational referral) could lead to poor health outcomes in terms of delays or even avoidable deaths [17–19]. There is, however, a lack of clarity on how accountability influences performance of health systems and maternal health outcomes.

There is gap in the conceptual and practical clarity on how accountability works in general [20, 21]. It is particularly because accountability is a contested concept as its connotations change with context and agenda. Further, the studies highlighting the role of accountability for poor maternal health outcomes lack a systematic approach particularly to analyzing how accountability problems lead to the outcomes [17–19]. Moreover, there is also a lack of a clear framework for such analysis. Due to its conceptual origin in disciplines such as political science, public administration, or ethics, the use of accountability concept in public health poses challenges [11]. Such an analysis would be crucial not only to fill the knowledge gap about the conceptual and the practical applications of accountability interventions but also to address the challenges regarding the attribution of such interventions (see Joshi [22] and the World Bank [14]). This article presents evidence from a *narrative and interpretive synthesis* approach of existing literature on maternal health and the health system in India—a country with one of the largest maternal deaths and inequities [1]—to analyze how accountability problems in the public health system could potentially contribute to maternal deaths and inequities.

### Conceptual framework

#### Links to existing accountability frameworks and developing a context-specific framework

**Fig. 1:**
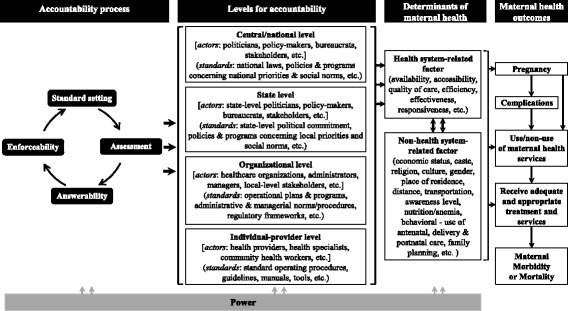
Framework to analyze issues of accountability

Studies in the health sector have used or proposed various frameworks on accountability [7, 12, 14, 23]. Some of the recent frameworks include the works of Molyneux et al. [6], George et al. [24], Van Belle and Mayhew [11], and Lodenstein et al. [25]. However, they all differ in scope (e.g., Van Belle and Mayhew—dimension of accountability) or focus (e.g., Molyneux et al.—community participation—and Lodenstein et al.—health providers’ response and social accountability) from the aim of this article. To support our analysis and interpretation, we developed a conceptual framework (see Fig. 1) integrating the two aspects, *accountability* and *maternal health*, based on the assumption that accountability potentially influences the performance of the health system, which could lead to poor health outcomes (in this case, maternal deaths and inequities) [17–19].

Given the diversity and applicability of accountability concepts, we chose to focus particularly on its political science, public administration and ethics dimensions, and the *institutionalist paradigm* as described by Van Belle and Mayhew [11]. In modern political discourse and democracies, such as India, accountability and governance of the public sector/institution carries greater weight since these bear the primary mandate to provide goods, including health services. Usually, this is achieved through political processes such as elections and legislation.

Accountability in the public sector in democratic states are usually based on the notion of delegation of authority or power to others or agencies—akin to the principal–agent relation [21, 26–28]. Citizens delegate authority to elected politicians or executives to carry out tasks on their behalf [11, 21, 26, 27]. The executives further delegate the authority to bureaucrats, administrators, and public officials down the chain of command. An unintended result of this delegation of power may result in its misuse against the principals/citizens. Therefore, in theory, accountability as a part of governance mechanisms generally aims to prevent misuse of power by holding agents at different levels in the health system to account for their action [13, 21, 28]. Command–control bureaucratic structures and horizontal accountability thus become a major accountability mechanism in the public sector. *Horizontal accountability* mechanisms operate within the state through internal checks and balances between different branches and levels of government, through which one state actor holds other state actors to account. This leads us to the *institutionalist paradigm*, which also identifies accountability based on the hierarchical relationships in a command–control bureaucratic structure [11]. It identifies accountability as a means to improved performance of institutions and organizations. Here, performance could be enforced through compliance with laws, rules, policies, and procedures, which we collectively refer to as *standards* in the framework.

These are not the only mechanisms; accountability in public sector is also ensured through *vertical accountability* mechanisms *or* non-state actors taking part in holding the state actors to account through activities such as elections, public hearings, or popular protests [21, 29]. Goetz and Jenkins [29] and Dasgupta [10] talk about *hybrid forms of accountability* where citizens/civil society engages with horizontal mechanism to ensure accountability of the public officials. Further, literature on *street-level bureaucracy* highlights the need for other forms of accountability that ensure the accountability of frontline public officials [30]. It is argued that public officials are also professionals who have certain degree of discretion and autonomy; it is difficult to ensure the accountability of such officials through the command–control bureaucracy approach that aims to limit their discretion by applying more rules, tighter control, and stricter procedures (see Hupe and Hill [30]).

We further integrated Joshi and Houtzager’s [26] performance assessment dimensions for accountability in our conceptual framework. In this approach, the *accountability process* involves four distinct cyclical steps [21, 31]: (i) *standard setting* or setting the behavior expected from agents (usually described in government policy documents); (ii) *assessment* of actual practices/performances of agents—such as individual patients, patient groups, non-governmental organizations (NGOs), or oversight bodies to assess whether they have met set standards/criteria; and the third and fourth steps are agents’ *answerability* for their performance and *enforceability* by relevant authorities, respectively.

To link our framework to specific maternal health outcomes, we added dimensions described in McCarthy and Maine’s model on the production of maternal health outcomes [32]. These include pregnancy, pregnancy-related complications, use/non-use of health services, and death/disability.

#### Application of the conceptual framework in India’s maternal health context

Accountability in the health system is about assessing the performance of the agents (also often called *actors*) with respect to set standards irrespective of their level [19, 33]. An overview of the chain of command in the Indian health system is provided in Fig. 1 (levels of accountability), which also includes individual health providers at the end of the chain. Accountability in the health system would involve assessing the performance of all *actors* at different *levels of the health system*, like politicians; bureaucrats; administrators; planners and decision-makers at the country/national, state, and sub-state government levels; and individual health workers against the *set standards*, *obligations*, *and performance targets* (Table 1).

**Table 1:** Standard for maternal health in India: expected behavior, obligation, and responsibilities

*Legal standards:* The Indian Constitution, the National Health Bill of 2009, and national policies and programs have guaranteed maternal survival as fundamental human rights and the government’s obligation [40, 52]. Further, being a signatory of international human rights frameworks—for example, the Convention on the Elimination of all forms of Discrimination Against Women (CEDAW) (1979) and the International Conference on Population and Development (ICPD) (1994)—the Indian government is obliged to realize timely and non-discriminatory access to appropriate maternal health services, irrespective of all racial and economic background, and to adopt necessary policy, legislative, budgetary, and administrative measures to ensure those entitlements [41, 93, 94]. The legal provisions of the Indian government like the Right to Information (RTI) Act and the Public Interest Litigation (PIL) also guarantee fundamental rights, including health [19, 41, 52].
*Policy standards:* Maternal mortality reduction has been a commitment of the Indian government since 2005 through the launch of Reproductive and Child Health Program-II and the National Rural Health Mission (NRHM) [75]. It has specifically emphasized equity in maternal health through a fully functional and accountable health system that particularly reaches the rural and the socio-economically vulnerable groups. Further, it also entitles free maternal health care through initiatives like the *Janani Suraksha Yojana* (*JSY*) and the *Janani Shishu Suraksha Karyakram* [36]. Similarly, state-specific programs to aid women to use maternal health services, for example, *Chiranjeevi Yojana* [95] and *Thayi Bhagya Yojana*—public–private partnership schemes for poor women in Gujarat and Karnataka, respectively, also are the obligation of the states. Besides, the Indian government mandated maternal death reviews to reduce maternal deaths by addressing service-related factors at all levels of maternal health care [38].
*Performance standards:* The NRHM identifies community-based monitoring, surveys, and internal monitoring to ensure accountability of the health system against the standards or services guaranteed. It has also emphasized the involvement of *Panchayati Raj Institutions*—locally elected representatives; *Accredited Social Health Activists* (*ASHA*); *Village Health*; *Sanitation and Nutrition Committee* (*VHSNC*); and *Rogi Kalyna Samiti*—a primary health center-level committee, self-help groups, and community-based organizations in monitoring and accountability of the health system, besides planning [36]. The NRHM has also laid out standards in terms of goals, targets, strategies, plans, operational guidelines, tools (e.g., International Public Health Standards), citizen charters, etc. for maternal health at various levels of the Indian health system. Further, clinical and disease management guidelines developed by the health ministry, respective disease control and management centers, health professional bodies, and the World Health Organization also serve as standards for performance for health professionals (e.g., doctors, nurses).

The *accountability process* in the health system has direct influence on *health system-related factors* of maternal health, which is also influenced by *non-health system-related factors*. While the outcome of maternal health could be depicted as a linear process, the process of accountability is an ongoing cyclical feedback process among the actors involved. Power is a major aspect of all accountability relations, which is highlighted by most of the accountability frameworks [7, 14, 24]. We identify power as a major structural determinant that has influence at all levels of health system and some of the factors not related to the health system, and throughout the accountability process.

## Methods

We conducted a scoping review of the literature from India to analyze how accountability problems in the health system contribute to maternal deaths and inequities. We searched for literature published after 2005 with PubMed and ScienceDirect using combinations of free terms (maternal health, health system, accountability, governance, and India) in the title, abstract, and key words (Appendix 1). The year 2005 was taken as reference year, since it is when the National Rural Health Mission (NRHM) was launched. The NRHM particularly restructured and strengthened the Indian public health system through financial, institutional, and management reforms [34]. The programs for reproductive and child health that were initially fragmented, inconsistent and vertical were also integrated into the NRHM [35]. Further, accountability was explicitly given due importance to improve health system performance in the NRHM [36]. This is not to say that accountability was not a focus before 2005. We also searched for additional articles by manual reference checking of review articles identified during the search.

### Inclusion and exclusion criteria

We followed the Joanna Briggs Institute reviewer’s manual [37] for inclusion of articles based on population, concept, and context—*population*; we included studies from India: *concept*, studies related to health system performance in relation to maternal deaths or access to/use of maternal health services, and *context*, the public sector. While issues of quality and performance are also reported in private health sector [38], which account for more than half of all institutional deliveries in India [39], we limited our study to the public sector, with which the obligation to prevent maternal deaths and address issues of maternal health inequities in India primarily lies [40–42]. We limited our study to empirical qualitative and mixed-methods studies to enrich our interpretative narrative synthesis as most quantitative studies are usually limited to variables that could be measured pragmatically and the variables that are usually included in related large-scale surveys such as those cited here [43–48]. We excluded reviews and studies related to maternal morbidities, other aspects of maternal health (e.g., abortions) and maternal health interventions (e.g., cesarean sections), or when full-text articles were not available or accessible.

#### Data extraction and analysis

We undertook a narrative and interpretative synthesis approach (see below) to analyze how accountability problems in the health system could potentially contribute to poor maternal health outcomes, such as maternal deaths and inequities. We conducted this in two steps.

##### Step 1

We extracted and analyzed data to provide a narrative summary of the causes of maternal deaths in India using Thaddeus and Maine’s “three-delay model” [49] (Table 2). We followed an iterative process of reading all included articles thoroughly, coding them based on the codes derived from the three-delay model.

**Table 2:** The three-delay model

A “three-delay model” developed by Thaddeus and Maine (1994) [49] is widely used to explore factors associated with maternal deaths. Management of obstetric complications is key to reducing maternal mortality where direct obstetric causes constitute majority of maternal deaths. The model is based on the fact that such deaths could be prevented through timely medical interventions. Focusing on the period between onset of obstetric complication and its outcome, it identifies “delay” as a pertinent factor contributing to maternal deaths: delay in decision-making to seek care as the *first delay*, delay to reach a health-care facility with appropriate care—the *second delay*, and delay in receiving adequate care—the *third delay*.	

##### Step 2

We undertook an interpretive synthesis approach to analyze the evidence generated from step 1 from an accountability perspective based on the conceptual framework we developed (Fig. 1). Dixon-Woods et al. explained that interpretive syntheses not only building on existing evidence but also applying new conceptual forms can generate new theoretical conceptualization and better understanding of the phenomenon of subjects posing methodological and conceptual problems [50]. The analysis and interpretation involved *lines-of-argument synthesis* approach, as described by Dixon-Woods et al. [50] and Campbell et al. [51]. We grouped the data on issues related to accountability at different levels of Indian health system based on the conceptual framework. We further interrogated and analyzed the result on their influence on the determinants of maternal health identified in step 1. We also drew on the work of Kaur [52] and other literature on accountability for concepts/theories on accountability to guide the interpretation and analysis. The use of interpretations and explanations from the included literature helped to ensure the “meaning in context” (see Weed [53]), while the use of the framework, the concepts/theories on accountability, and the case helped to generate “synthetic constructs” (see Dixon-Woods et al. [50]) and validate our arguments on the mechanisms of influence.

## Results and discussion

### Overview of the evidence base

**Fig. 2:**
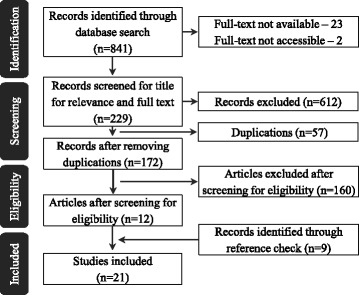
Study flow chart

We included 21 articles for our study (Appendix 2): 12 identified through database search and nine through reference check (Fig. 2). The articles included studies from 16 Indian states conducted between 2002 and 2014. Nine studies explored factors contributing to maternal deaths [17, 18, 52, 38, 42, 54–57], eight were related to factors influencing access to use of maternal health services [19, 58–64], two explored policy contexts [3, 65], and two were related to the implementation of maternal death review (MDR) [66, 67] (see Appendix 2 for detail).

In the following sections, we first present the narrative summary of causes of maternal deaths in India based on the three-delay model. Then, we present the results of the interpretive synthesis based on the accountability process of the conceptual framework: gaps in standards, performance of health system, and answerability and enforceability at different levels of health system. The performance section includes the implementation of the standards as a result of our assessment, rather than the assessment as an accountability function. The result of the assessment as accountability function is presented under answerability as gaps in generation of information. We also discuss their implications on maternal health outcomes.

#### Causes of maternal deaths in India

##### First delay

Studies from six Indian states conducted during 2009–2011 reported the first delay in 35–60% of maternal deaths [42, 55, 66, 67]. Lack of awareness among women and their family members of pregnancy-related risk factors or the value of institutional delivery and perceived inability to afford treatment and transport to health facilities were the major reasons mentioned for such delays [38, 42, 54, 61, 63, 64]. Women lacked information, particularly on the danger signs of pregnancy, birth preparedness, and emergency preparedness [38]. In Jharkhand, it took households from 2 to more than 7 days to recognize obstetric complications leading to death in 90% of the cases [56]. Studies reported that in extreme situations of financial hardship to meet treatment and transport expenses, families avoided seeking any treatment [19, 38, 54].

Other reasons for delays are the social perception of pregnancy and childbirth as a normal phenomenon rather than a life-threatening situation [54, 64], relating pregnancy-related risks to supernatural etiology [62], negatively perceived and/or experienced quality of care at public health facilities [42, 60, 63, 64], and fear of medical procedures and disrespectful care or unfamiliar environment at health facilities [58, 62, 63]. Women’s low status, including in decision-making, was also reported as a cause for delay in seeking health care in rural areas of Madhya Pradesh, Odisha, and Kerala [42, 55, 62].

##### Second delay

Once the families made the decision to seek health care, studies reported delay in reaching a health facility in about 20–50% of maternal deaths in 12 Indian states [38, 55, 57, 66, 67]. Almost all cases of maternal death in rural Madhya Pradesh experienced the second delay [42]. Such delays were mainly due to financial problems [18, 58, 64] and physical accessibility such as lack of transport [18, 38, 42, 57, 58, 61, 64] or roads, or difficult geographic locations [19, 38, 54, 55, 62]. It took up to 5 h or more to arrange transport in one third of maternal deaths in Jharkhand [56]. About 60% of the families had to borrow money to meet health-care expenses in rural Rajasthan [54]. Geographic access in terms of long-distance travel and difficult terrain was the cause of maternal deaths, especially for tribal women in rural Kerala [55].

##### Third delay

About 15–60% of maternal deaths were due to delay in receiving appropriate health care in 13 Indian states [18, 38, 42, 55, 57, 66, 67]. The third delays were primarily due to health system-related factors, mainly in terms of availability and quality of care.

Studies reported the lack of fully functional obstetric care at designated levels of health care in India in terms of limited infrastructure, health specialists, and equipment and supplies, including blood and medicines [17–19, 38, 42, 56, 58, 62, 63]. There was 60% shortfall of primary health centers (PHCs) responsible for providing basic emergency obstetric care in communities and 22% shortfall of sub-centers responsible for providing basic maternal health services along with awareness through home visits by auxiliary nurse-midwives (ANMs) in Jharkhand [56]. Even district hospitals and community health centers that are regarded as first-referral units were often reported to lack the necessary health specialists, nurses, and ANMs due to vacancies or staff on long leave or training [18, 19, 38, 56]. Even when they were available, they were often deployed to other programs such as polio, sterilization, or administration [18, 19, 38]. The available staff also lacked skills to identify and manage obstetric complications in a timely and appropriate fashion, which led to delays in health care [17–19, 38]. Lack of blood at designated health facilities or blood storage units emerged as a critical problem that led to delayed or inadequate blood transfusion in emergencies [17–19, 38, 42, 54].

Lack of proper immediate postnatal care (PNC) contributed to a large number of maternal deaths in India. Studies reported PNC to be completely absent both at health facilities and communities [17–19, 38, 54], despite the high prevalence of maternal deaths during the postpartum period (48–84%) [38, 54, 57, 66], most occurring within 24 h after delivery [38].

The lack of services often led to a high number of referrals in India, often multiple, of women with obstetric emergencies that led to further delay in care [18, 19, 38, 42, 54, 56, 58, 66]. Studies reported multiple referrals in 37–59% of maternal death cases [17, 38, 42, 55–57] and as high as seven referrals in some cases of maternal deaths [17, 38]. The multiple referrals were either because families took women to more than two health facilities (due to lack of information or not being satisfied with the care provided) or they were referred by health-care providers [42].

An overview of the determinants of maternal deaths and maternal health inequities in India based on the “three delay” model is presented in Table 3.

**Table 3:** Causes of maternal deaths in India

First delay	- Lack of awareness (obstetric risk factors, danger signs, or value of institutional delivery) - Lack of antenatal care (opportunity to provide information on obstetric risk factors and maternal health care) - Cost constraints—related to poverty (treatment and indirect costs, travel, perceived affordability, corruption and informal payments at health facilities) - Social norms considering pregnancy and childbirth as a normal phenomenon - Gender or women’s low status - Perceived and/or experienced quality of care - Fear of cesarean section, surgery, disrespectful behavior, and unfamiliar environment at health facilities
Second delay	- Cost constraints (to arrange money) - Lack of roads - Lack of transport, including difficulty in arranging transport at night - Lack of information (regarding availability of services) - Geographic location (e.g., tribal settlement in remote areas) - Difficult terrain (steep hill and water-logged road during rain) - Referral(s)
Third delay	- Non-availability of services (designated obstetric services at different levels of health system, abortion, and postnatal care) - Lack of health professionals, including lack of competency or skills among health professionals - Lack of drugs and supplies, including blood - Cost constraints (to pay for drugs, blood, and treatment)

There were multiple delays rather than one particular delay in most maternal death cases [19, 38, 55]. Further, in many cases, multiple determinants interacted to produce the adverse maternal health outcomes [19, 38]. For example, the negative reinforcement between multiple referrals and issues of travel, which were again associated with cost, time, and distance of up to 100 km [19, 54], exacerbated poor access to health care [19, 42, 57]. In Madhya Pradesh, 13 of the 26 women treated at a district hospital were referred to higher facility in a city about 4 h away by road; they could not afford to make the journey and consequently died in the same hospital [18].

The majority of deaths in 10 Indian states (77–84%) were among women who sought care—either at health facilities, during facility-to-facility referrals, while returning from a health facility or at home after returning [38, 57]. In rural Rajasthan, the women who sought care but died at home had been provided only outpatient treatment, were discharged prematurely from hospital, or brought home against medical advice because their families could not afford further treatment [54]. These studies highlight the overarching influence of health system-related factors over those not related to the health system for maternal deaths and inequities in India.

The “Obstetric transition” model developed by Souza et al. (2014) [68] categorizes India under “stage III,” characterized by a high maternal mortality ratio (MMR), variable fertility, predominantly direct obstetric causes of maternal deaths, and issues of access to health care for some populations. More than 80% of maternal deaths in India are due to direct obstetric causes [69]. In such a situation, access to and quality of health care remains crucial to achieve a significant reduction of maternal deaths [68].

#### Analyzing determinants of maternal deaths using accountability lens

The health system not only can address the issues of access to and quality of health services, including maternal health, but can also potentially address other causal factors such as transport, geographical and social barriers through interventions such as intersectoral actions, empowerment, and social support [70]. Examples from Sri Lanka and Malaysia have shown that a reduction in maternal deaths and improvements in maternal health equities can be achieved through sustained policy interventions driven by strong political commitment and coordinated concerted efforts to reach disadvantaged groups of women to provide quality essential obstetric care [71]. Effective interventions include management of obstetric complications, and addressing all possible determinants of maternal health inequities, along with the capacity of the health system to implement these [3, 71]. Further, the overall improvement in maternal health is dependent on the function of entire health system rather than single interventions [3].

A large number of maternal deaths and maternal health inequities in India (Table 4) show not only the Indian government’s unfulfilled commitment to respect, protect, and fulfill women’s maternal survival rights but also a lack of accountability of the health system. A maternal death case from Haryana also supports this (Table 5)*.*

**Table 4:** Maternal health inequities in India

Even though the maternal mortality ratio (MMR) has reduced in India, from 556 (per 100,000 live births) in 1990 to 174 in 2015 [1], the country has one of the largest number of maternal deaths in the world. It is far above the national commitment to achieve the Millennium Development Goal - 5 target, i.e., to reduce the MMR by three quarters or 139 by 2015 [96]. An estimated 45,000 maternal deaths took place in India in 2015, accounting for about 19% of the global number [1]. Further, there are large intra-country disparities in MMR (maternal deaths per 100,000 live births): higher among the northern states such as Assam (328), Uttar Pradesh (292), and Rajasthan (255), and lower among the southern states such as Kerala (66), Maharashtra (87), and Tamil Nadu (90) [97]. Women, particularly from rural, poor, and certain castes, such as scheduled castes/scheduled tribes (*SC/ST*), continue to die from *avoidable* maternal causes across all Indian states [38, 67]. More than 70% of the maternal deaths in 10 Indian states were among women from *SC/ST* [38], while *SC/ST* constitute about 25% of the total population in these states [98]. The use of maternal health services is also particularly low among the rural, the poor, and women from the marginalized castes [99]. The latest available survey data show that institutional delivery was higher among urban (70.4%) than rural (37.8%); *other* castes (58.9%) compared with ST (32.5%), SC (41.9%), and other backward castes (OBC) (47.8%); and richest (highest wealth quintile) group (80.1%) compared with poorest (lowest wealth quintile) group (19.1%) [99].

**Table 5:** Case example

Kaur [52] referring to Shanti Devi’s litigation case from Haryana state highlighted that flaws at different levels of health system from policy to service provision led to her death. She died of postpartum hemorrhage in her sixth pregnancy after a home delivery without any medical assistance. After the Delhi High Court intervened for the investigation of her death, it was identified that she died of an obstetric condition that was preventable and the health system was particularly responsible for her death for not acting responsibly to provide services guaranteed by the state and addressing her socio-economic constraints (see [52]). It particularly highlights the lack of accountability of the health system to ensure functional health-care services and interventions to address the determinants of their access.	

In the previous section, we identified the causes of maternal deaths in India. In the following section, we present the accountability issues, based on the accountability process of the conceptual framework, at various levels of the Indian health system, and discuss how they relate to or what implications they have on the causes identified in the previous section. We particularly discuss the issues as gaps in standards, performance, and accountability functions, i.e., answerability and enforceability.

### Gaps in standards and their implications

We observed gaps in standards mainly in terms of policy gaps or conflicting/discriminatory policies and political commitment. Within the premise of principal–agent relationship, these are examples of political accountability. While political accountability, in broader sense, can be applied to the conduct of all public officials, in narrow sense, it can be applied to politicians and policy-makers [21]. In that sense, at policy levels, it is concerned with the *appropriateness of policies* (standards) and *policy-making process* (performance) relating to how the politicians and policy-makers honor their electoral promises, policy commitments, and health service delivery targets [12, 13]. In the health sector, it is also concerned with how such policies respond to social needs and concerns, and norms and issues of equity.

#### Policy gaps

Studies reported gaps in or failures of national health policies to ensure continuous and non-discriminatory access to appropriate maternal health services to all sections of population. Even though the NRHM has been successful in increasing institutional deliveries and reducing inequities in maternal health service use through a cash-based incentive program—the *Janani Suraksha Yojana* (JSY)—studies show that it has not been able to effectively reduce the MMR [59, 72–74]. It has been criticized for pushing for greater institutionalization of childbirth, but not giving equal attention to ensuring quality or institutional readiness to handle the increased maternity workload and continuity of care [18, 38]. The exclusive focus on institutional delivery has particular implications as it has led to increased number of women presenting at health institutions for childbirth, but the health system has not been strengthened at the same pace to ensure “safe” deliveries [18, 19, 38], hence contributing to maternal deaths taking place at health facilities, during facility-to-facility referrals, or on the way back home.

The exclusive focus on institutional deliveries hides the need to ensure attention to “continuity of care” before, during childbirth, and in the postpartum period. For example, studies reported a large number of women not receiving any antenatal care (ANC), and for those who did, it was limited to providing iron and folic acid tablets and tetanus toxoid injections [18, 19, 38]. Women in India lacked information on obstetric danger signs and maternal entitlements also because of lacking or inadequate ANC [38]. ANC visits are a space for providing pregnant women and their family with information and counseling on birth preparedness and emergency readiness. Further, it is also crucial in screening certain health problems such as anemia, eclampsia, sickle cell anemia, malpresentations, and their timely management [38]. So, policies’ equal attention to ANC and PNC could have prevented most of the maternal deaths related to lack of awareness or health problems and those taking place in immediate postnatal period.

In many states, the JSY benefits or maternal entitlements are not considered for pregnant women below 19 years of age, women with more than two children, migrants, and married women who are not residing in their husband’s home [19, 38]. The discriminatory policies not only directly impeded such women from accessing maternal health services during emergencies but also had implications on the recording and reporting system. Health workers mentioned not recording and reporting pregnancies and deaths of women not eligible for the JSY benefits [19].

#### Political commitment

Political commitment relates to sustained attention or priority to maternal health issues, and taking effective actions to address the issues in terms of financial, human, and technical resources and social determinants of maternal health [75–78]. Under the Indian federal system, health is a matter for states, which have the responsibility for implementing national health policies, including the NRHM [75, 65].

Despite a strong political commitment to maternal health from the national government, this is not matched at the state level. This is clear from the differences in maternal mortality and especially in the northern states where the majority of India’s maternal deaths occur [79]. Studies have extensively reported limited infrastructure, equipment and supplies, and inadequate and incompetent human resources responsible for the poor quality or lack of health care and referrals [18, 19, 38, 59]. In Tamil Nadu, the low MMR and maternal health inequities were the result of the state government’s strong political commitment to improving infrastructure and ensuring adequate training and deployment of health professionals, management, monitoring, and accountability [19, 65, 76]. It suggests that the poor situation of maternal health in the northern states is particularly due to lack of political commitment.

Some gaps in standards were also observed in terms of unclear guidelines and the absence of state level policies and sub-state or district-level plans. A study reported that the Indian Public Health Standards guidelines are unclear about the number of drivers to be hired per ambulance, leading to unavailability of ambulances during emergencies due to the lack of drivers [57]. The lack of a well-designed referral protocol was reported in rural Kerala, which led to irrational referrals [55]. Studies also mentioned that despite the high prevalence of anemia and malaria, district plans lacked specific programs to address them [18, 38].

### Gaps in performance and their implications

Policies do not automatically translate into action; implementation and contextual factors that influence implementation are particularly crucial [3], such as representative politics (policy-making), capacity of the overall health system, community participation, administrative challenges, and issues with health professionals’ performance.

#### Representative politics

A study in Uttar Pradesh highlighted that women, especially the poor and the marginalized, lack any voice in policies due to the issues of representation owing to their limited capacities and access to resources, resulting in the skewed distribution of health services [10]. Even when such women succeeded in gathering concerns about maternal health and raising collective voices, such voices had limited impact and had little influence on policy decisions at state and national levels that most affected them.

Solar and Irwin [70] considered that issues of representation and voice are particularly due to the issue of power asymmetry between the poor and the marginalized groups and the dominant or socioeconomically advantaged groups in terms of lack of capacity and resources to influence decisions. The dominant groups influence the agenda of public debate and decision-making to achieve their strategic goals. Such power asymmetry ultimately shapes social hierarchies along the line of power of the dominant/advantaged groups generating social inequities, including health inequities. We recommend further studies on influence of power asymmetries on social hierarchies, health systems, and maternal health outcomes.

#### Health system incapacity

A study from Gujarat critically highlighted the issue of lack of capacity of the health system to implement maternal health interventions [3]. The health system lacked capacity particularly in terms of being solely dependent on individual stakeholders and not following structures and processes like evaluation and follow-up, and coordinating various interventions and actors at various levels of the health system and outside. The dependence on individual stakeholders has implications for losing focus and momentum in implementing policy as well as losing long-term memory and lessons learned to improve policy implementation. The incapacity to coordinate particularly hampers the health system in achieving the national goal of equity in maternal health through convergence and decentralization [3]. The capacity to implement health policies is also influenced by socio-political contexts in terms of political commitment, and political inconsistencies and conflicts between governing institutions or agencies [65].

#### Community participation and capacity

To achieve equity in health, including maternal health, the NRHM has particularly emphasized the role of community participation in strengthening health system through convergence and decentralization. It has especially highlighted the participation of various stakeholders at district level and below to plan, manage, and monitor health programs and make the health system accountable through committees like *Rogi Kalyan Samiti*, a peripheral decision-making health unit; *Village Health Sanitation and Nutrition Committee* (VHSNC); and community mobilization through community health workers, the *Accredited Social Health Activists* (ASHAs) [36]. However, studies reported issues and challenges with the performance of such committees, especially the VHSNC. Even though composition of the VHSNCs met the standard to include women, socially disadvantaged groups, *Panchayati Raj Institutions*—locally elected representatives and self-help groups, these members lacked knowledge about their roles and responsibilities due to lack of formal training [80–82]. Therefore, their participation in regular meetings and preparing village health plans representing communities’ voices were limited [80–82]. While ASHAs were able to perform the role of “link worker” and “service extension” in maternal health, their performance related to mobilizing the community in local health planning and ensuring the accountability of existing health services were limited due to their limited understanding of their role as health activists [83].

#### Administrative challenges

Studies reported issues with the implementation of national policies due to policy norms and administrative challenges. For example, in many states, the JSY requires documentary proof of poverty (e.g., *Below Poverty Line* (*BPL*) cards). However, studies reported problems with such cards often not being issued. Further, obtaining such cards is beyond the capacities of marginalized groups such as migrants and the poor [19, 52]. Studies also reported that women faced challenges in benefiting from the JSY program due to the requirement to open a bank account, the cost of which was reported to be equivalent to the amount of the JSY payment; receiving the funds as they have to wait many hours to receive the check or have to pay large bribes to hospital staff to receive their payment; lacking the required identification for processing the JSY payments [58, 63]; and the cash payment being less than the expenditure in terms of both monetary and real costs [58, 64].

#### Health workers’ performance

Studies frequently highlighted issues with professional accountability—assessed against ethical standards of professionalism [13]—of health professionals responsible for maternal deaths in terms of not discharging designated duties, showing negligence in providing health care, making inappropriate and irrational referrals, inadequate interpersonal communication, behaving in a demeaning fashion towards patients, and corruption and demanding informal payments [18, 19, 38, 42, 55, 58–60, 62, 63].

The ANMs based at PHCs and health sub-centers in Madhya Pradesh were not visiting villages to provide ANC as they were supposed to do [18]. Health professionals were reported not even attending women presenting for obstetric emergencies at health facilities in some cases [38]. Despite having a well-equipped operation theater at a district hospital, health professionals in Madhya Pradesh were reported not conducting emergency operations at night [18]. Health professionals were also reported not adhering to protocols at all levels of care, including in the administration of drugs and treatment [18, 38].

Studies mentioned that health staff either ignored or showed negligence in immediately providing health care, treating obstetric complications and during referrals [38, 42]. During referrals, health professionals often did not stabilize women before referring them contributing to deaths en route or soon after arriving at the referred facility [18, 55]. Limited interpersonal communication between health professionals and their patients [58] and issues with communication, for example, not presenting blood-test results [38] or reasons for referrals [42] to the patients were also reported.

Demeaning behavior in terms of verbal abuse such as using foul language and physical abuse such as slapping and beating of women during delivery were frequently reported in most Indian states [18, 19, 38, 42, 55, 58, 60, 61, 63]. A study in Madhya Pradesh also reported health facility staff using coercion, for example, forcefully holding women’s legs apart during labor [59]. Such behaviors occurred particularly among the women from the poor, rural, and tribal communities and with high parity [18, 59, 60, 62].

Despite the provision of free maternal health-care services at public health facilities, health providers were reported making illegal demands for money for check-ups, diagnosis and treatment, medicines, blood, and services such as cutting the umbilical cord, cleaning, and ambulances that were supposedly free [18, 19, 38, 42, 58, 63]. Corruption was common in health facilities [18, 63], and respondents were either treated badly or denied care if they were not able to provide financial tokens to health providers [63].

Such unaccountable behaviors of health providers were partly due to their indifferent attitudes and partly due to structural problems of the health system and the asymmetrical power relationships between health-care providers and the patients. Health-care providers were sometimes unable to pay enough attention to women in emergencies and were reported to be extremely stressed and lacking motivation due to understaffing and over-pressured health system [18, 38, 63]. Reasons reported were inadequate incentives and lack of institutional recognition [58]. Nurses, ANMs, and ASHAs wasted too much time or failed to recognize complications due to lack of knowledge since they had inadequate training and supervision [18, 38]. The ANMs based at PHCs and health sub-centers were reported not visiting villages in rural Madhya Pradesh due to lack of roads and transport [18]. The lack of blood—one of the reasons for multiple referrals—was due to non-availability of donors, non-availability of blood from the required blood group, and sometimes due to mismanagement at the blood-storage units [42]. Health providers in Uttar Pradesh justified that their demeaning/inappropriate treatment towards women were because of heavy workloads and overcrowding [58].

The disrespectful and discriminatory behaviors of health-care providers towards disadvantaged women were due to the asymmetrical power relations, affecting information, expertise, and power to determine access to health services [7, 12, 84]. Health-care providers usually know more about health and health care than their patients, putting the patients in a dependent and vulnerable position in a patient–provider relationship [84]. Despite standard procedures, providers can exercise significant gatekeeping power, for example, determining who receives what care and how [12, 84].

Further, power also influences health workers’ attitudes and behavior towards disadvantaged women by translating broader social values and norms of looking down on the poor and marginalized, and women in general, into health system [7, 10, 85]. This is evident in terms of the discriminatory behavior of health workers. Two studies from Uttar Pradesh and Madhya Pradesh also mentioned that women indicated that their disrespectful treatment at health facilities by health providers was largely due to the issues of social and economic status [59, 63]. Moreover, the health providers view the (in)capacity of such women to demand good quality and respectful services leading them to provide them poor care [10, 85]. Such social values and norms also have implication for such women—they do not see themselves as genuine right holders and do not claim their rights [10]. Studies described situations where poor women and their families were reluctant to face repercussions demanding accountability and thus appeared helpless and either accepted such behaviors silently rather than raising voice against such behaviors or preferred not to seek care at all [59, 60].

The indifferent attitudes and behaviors of health professionals had implications for women’s perceived and/or experienced quality of health care at public health facilities. Several studies highlighted women’s perception and experience of care as a reason why they were reluctant to seek care at health facilities or non-compliance with care in India [60–63, 86]. For instance, women perceived referrals were unnecessary as health professionals did not tell them why they were being referred [42]. For many women in Uttar Pradesh who delivered at home, prior experiences of family members and neighbors shaped their perceptions and influenced their decision to not to seek care because they were mainly concerned about the way they would be treated at health facilities, specifically fear of being disrespected, ignored, or treated poorly [63].

On the other hand, courteous, empathetic, and supportive behavior by health workers has a positive impact on women’s use of maternal health services. Frequent visits and support from the ASHAs motivated women to switch their preferences from giving birth at home to going to a health facility for childbirth in three Indian states [58, 64]. Similarly, a willingness to respond, friendly behavior, politeness, respect, and emotional support provided by all staff at PHCs were the reason why women preferred to give birth at the PHCs rather than in private and higher-level public health facilities in Tamil Nadu [87]. For women, this mattered more than the technical competence of the providers [87].

Our findings on health workers’ performance echo the work on *street-level bureaucracy* [30, 88] and findings of Topp et al. [89]. The street-level bureaucracy approach asserts that the patterns of practice by frontline public officials are influenced by a wide range of contextual factors in which they work such as shortage of resources, their interaction with individual clients, their micro-network, and web of multiple relations. Such patterns of practice also unintentionally and informally shape policies [30]. In their study, Topp et al. discussed the fact that structural constraints such as limited material and human resources influence providers’ personal choices and actions, which further influence service quality and responsiveness [89].

### Gaps in accountability function: answerability and enforceability

Accountability functions basically refer to ensuring the answerability and enforceability of a health system. We observed gaps in these functions in terms of information and sanctions in the Indian health system.

#### Answerability gaps

Key to ensuring answerability is generating information through the performance assessment and monitoring system. However, studies reported that the health system lacked proper regular monitoring of policy implementation and health services [3, 17–19, 38]. For instance, a study reported the lack of monitoring of national policy implementation at the state level by the central level [19]. A lack of proper mechanisms to gather information at the district level on where, when, and why deaths and injuries occurred was reported in Uttar Pradesh [19]. Lack of documentation in several cases of referrals was reported in the district hospital in Madhya Pradesh, due to which the outcomes/status of the cases were not known [18]. It highlights issues with lack of follow-up, since once women were referred, there was no follow-up to ensure what happened thereafter [18, 19]. Challenges with the monitoring system included the inability to coordinate existing parallel monitoring systems and lack of appropriate indicators and confidence in data due to inconsistencies in the way they are collected and analyzed [3].

Maternal death review (MDR) is considered a powerful tool for accountability and to monitor implementation and evaluate the effectiveness of health care, especially at district level. However, despite the Indian government’s mandate to conduct such reviews, these were not carried out effectively at any level of the health system in most states [18, 19, 38, 66]. For instance, in 10 Indian states, health teams made enquiries in only 40% of the maternal deaths [38]. Under- or non-reporting of maternal deaths was also reported from PHCs, tribal communities, and those related to illegal abortions and taking place in early antenatal period [66]. Possible reasons for the under- and non-reporting were fear of punitive action, poor continuity of care, lack of clarity and priority among health workers to register and report maternal deaths, etc. [18, 19, 66].

Moreover, mechanisms like grievance handling or redressal were often lacking, for instance in Uttar Pradesh and Madhya Pradesh [17–19, 38, 58]. Women faced obstacles in filing complaints due to lack of awareness of their entitlements, absence of a clear complaint procedure, poor access to any complaint procedure, absence of a response mechanism, and fear of reprisals by doctors and health workers [18, 19]. Instances were also described where even ASHAs also faced reprisals from staff nurses when they wanted to complain about staff nurses’ illegal demand for money from women seeking health care in Uttar Pradesh [19]. Further, health officers often denied complaints about issues with health services [19] or avoided dialog when complaints were filed [18].

#### Enforcement gaps

Sanctions are critical for enforcing standards [9] and improving providers’ responsiveness [22]. However, studies reported issues with sanctions in the Indian health system particularly with the horizontal accountability mechanisms. Horizontal mechanisms such as administrative control, performance assessment, and disciplinary procedures, even if they exist, may not be properly enforced mainly due to the lack of sanctions set out in the policies, and asymmetrical power relations in the hierarchical health system. There appeared to be a lack of clear sanctions set out in national regulations or guidelines against bureaucrats who fail to act [19]. One of the reasons the Indian government failed to effectively implement the Right to Information law was lack of sanctions in the legislation against bureaucrats who denied access to information [19]. Further, in a study in Karnataka, George mentioned that administrators also lacked administrative authority to impose sanctions, thus limiting the effectiveness of the internal control mechanism in the health system [9].

In circumstances of misdeeds or inappropriate health care, sanctions are often imposed on staff at lower levels of the health system [10, 17, 18, 60, 66]. George highlighted that internal control mechanisms like disciplinary actions in Karnataka were often jeopardized by misuse of power by higher authorities demanding money, through corruption, political interference, or personal relationships between the officials through informal norms and political leverage [9]. The higher authority often saw the disciplinary mechanisms as an opportunity to earn money through corruption or to use lower-level health staff as scapegoats to protect their reputation [9].

Joshi [22] highlighted that information does not necessarily lead to accountability; there needs to be pressure or incentives for the public authorities to respond. Lack of sanctions or providers’ response also demotivate those using public services to make complaints against public officials [60, 90], opting instead to withdraw from using public services [60]. Further, the repercussions and reprisals that the service users and lower-level staff face also discourage them from lodging complains against public officials, including senior public officials [60, 90].

While accountability can function both as control and constructive mechanism to improve the performance of the health system [9], lack of accountability can potentially lead to poor performance. The gaps in standards and performance or implementation of standards in the Indian health system potentially relate to the gaps in accountability functions, both answerability and enforceability. For example, policy-makers lacked information on progress and obstacles to policy implementation critical for effective policy-making, thus resulting in policy gaps. Due to the poor implementation of the MDR, decision-makers and planners lacked information on where, when, and why women die during pregnancy, childbirth, or in the postnatal period [18, 19, 38]. As a result, district-planners lacked critical information to address problems at the district or local level. The lack of implementation of community monitoring under the NRHM in Odisha state was due to lack of enforcement by the national government and lack of follow-up by district-level authorities and administrative officers [91].

Further, information generated through the performance assessment and monitoring system builds up evidence for generating awareness, which are crucial for prioritizing problems, initiating actions, and sustaining them [71, 92]. Awareness of the magnitude of maternal deaths was a major factor in generating political will to tackle the problem, which further led to a sharp reduction in MMR in Sweden [92] and Malaysia and Sri Lanka [71]. Maternal death audits built the evidence base to inform decision-makers at state and district levels, which helped to mobilize support and resources for improved maternal health services in Tamil Nadu [65]. Health-care providers’ performance assessments are crucial to hold them accountable, which was described as another factor necessary to improve the quality of care and reduce maternal mortality [92].

#### Discussion on study context and framework

This study is, according to our knowledge, among the first [19] to systematically analyze how accountability leads to maternal deaths and inequities in an era when accountability is being increasingly recognized as a crucial factor for improving the performance of any public service delivery systems. We conducted the study in view of the lack of empirical studies to analyze such a relation. Most of the existing studies focus on how accountability interventions work rather than how a lack of accountability leads to any problem [10, 25, 91].

The framework was able to gather evidence from different studies from India on maternal health, health system, or accountability and provide a comprehensive picture of accountability process at all levels of Indian health system and its influence on maternal health determinants and outcomes. The framework and the narrative and interpretive synthesis approach have been able to collate the existing evidence to interpret or explain the phenomenon of influence (e.g., accountability on the performance of health system and maternal health outcomes) in terms of cause and effect; for example, lack of services or neglect and indifferent attitudes of health workers led to multiple or unnecessary referrals, which further led to delay in women reaching and receiving appropriate care.

We developed a conceptual framework to analyze accountability issues in health system for maternal health in particular. However, we see the potential for its application for health problems, beyond maternal health (adjusted for the determinants and outcomes), in any health system beyond India (adjusted for the levels for accountability) or focusing on any specific level of health system (e.g., national, organizational, individual) or aspect of accountability (e.g., standard setting, answerability, enforceability).

We see the potential of the framework to capture other critical aspects covered by other accountability frameworks, such as street-level bureaucracy. Similarly, we have been able to identify all possible relevant accountability issues identified across the three axes described by George et al. [24]: *power*—for example, sanctions, monitoring; *justice*—for example, political representation, political commitment; and *ability*—for example, capacities, providers’ attitudes, inputs in terms of human resources, equipment, and supplies.

We also observed that this framework has the potential to identify and analyze issues related to other paradigms or all aspects in the comprehensive framework proposed by van Belle and Mayhew such as social, political, organizational, and individual dimensions [11]. For example, the influence of social values and norms on the attitudes and behavior of health workers towards women or of representative politics on health policies.

#### Limitations of our study

The first limitation relates to the limited number of studies, particularly on health system performance at the organizational level. There were also few available studies to provide a comprehensive picture for any specific Indian state. This might be due to the lack of such studies or the limitations of our study strategy (scoping review, and inclusion and exclusion criteria). However, we have been able to integrate evidence from different Indian states and provide a comprehensive picture at national level. We have also been able to highlight areas of potential influence and generate synthetic constructs of their influence to guide further studies. So, we recommend further studies contextualized to any specific Indian state, level of health system or context, and with a methodology to identify more and relevant studies.

Our analysis also has been limited to public sector while accountability issues have also been reported in the private sector, which accounts for a large proportion of maternal health care and maternal deaths in the country. More deliveries take place in private facilities (20.2% of all deliveries) than in public facilities (18.0%) [39]. Subha Sri and Khanna reported about 15% of the total maternal deaths took place in private facilities in 10 Indian states [38]. A strong point is that the studies included covered issues from 16 Indian states with poorest to the best maternal health indicators in India. We identified identical issues of accountability even in better performing southern states like Kerala.

We also faced challenges in establishing an explicit linkage of different accountability issues with adverse maternal health outcomes. This is because hardly any studies explicitly explored the linkage between factors and maternal health outcomes or used an accountability perspective. Therefore, we urge that more empirical studies be made on this topic.

A major limitation also pertains to the conceptual paradigm implicit in the framework, i.e., the *institutionalist paradigm* and the *command–control bureaucracy*. This might have resulted in identification and interpretation of the results predominantly in the context of the latter. However, we see the potential of the framework to include approaches beyond a command–control bureaucracy (such as vertical accountability) in analysis, especially by including articles on such approaches and particularly to understand how such approaches influence accountability processes.

Finally, the framework we proposed and the methodology we adopted have not be tested or used anywhere before. Through this framework, we have been able to use the limited information available on maternal health in the Indian context and connect threads to analyze and explain the influence of accountability on maternal deaths and inequities in India. However, we urge that further research test both the framework and methodology for further applicability, limitations, and challenges.

## Conclusion

In this study, we aimed to analyze how accountability problems in India’s health system contribute to maternal deaths and inequities. For this, we reframed the relevant comparable information from limited existing empirical studies on maternal health services at different levels of Indian health system from an accountability perspective through a narrative and interpretive synthesis approach. This enabled us to identify the hot spots of systemic flaws from the accountability perspective and systematically show that lack of accountability leads to maternal deaths and inequities.

There is a large overarching influence of health system-related factors on the availability, accessibility, and quality of maternal health services leading to maternal deaths and inequities in India. A potential link between such factors was identified with gaps in accountability functions at all levels of the health system. Gaps in standards pertained to policy gaps or conflicting/discriminatory policies and political commitments. A large number of issues concerned performance gaps or gaps in implementing existing standards in terms of health system incapacities, representative politics, and health workers’ performance. Underlying this is potentially a lack of adequately functioning accountability mechanisms at different levels of the health system.

Besides the gaps in different accountability processes, the study was able to develop some critical messages:

- First, maternal health outcomes such as maternal deaths and inequities are influenced by a wide range of factors not necessarily covered solely by the health and health system domain such as political, legal, and governance. These factors operate differently at different levels of health systems in interaction with each other to influence maternal health outcomes.
- Second, accountability at all levels of the health system are interconnected and influence each other. This leads to a crucial point that accountability should be viewed as a systemic problem. Studies have shown that accountability is often viewed as an individual responsibility and that it is often limited to frontline health professionals and providers, for example, health specialists, doctors, nurses, ANMs, and ASHAs [17–19].
- Moreover, accountability is often equated with blame and punishment, while the systemic perspective suggests that accountability practices should attempt to resolve problems constructively [17]. Accountability practice should assess performance at all levels of a health system—national, state, district, and individual—and essentially identify and rectify systemic flaws at all levels.
- Last, power is central to all accountability relationships and functions. So, as other studies have emphasized [7, 18, 70], any effort to address issues of accountability would require addressing the issues of power asymmetries. This would require addressing issues in the hierarchical power relationships in the health system and empowering communities and especially disadvantaged women to influence policies. At the same time, this would require duty bearers to change their personal attitudes, in particular to recognize disadvantaged women as genuine right holders.

We found that the general model of accountability helps us to explore the problem in the health system beyond service delivery to wider areas such as policy, political commitment, and administration. It highlights their relevance and importance in analyzing maternal health problems to provide a comprehensive picture of the factors of influence in order to address them in a more comprehensive and systemic way. This study has particularly highlighted areas of potential influence for accountability in maternal health and generated synthetic constructs on the mechanisms of their influence to guide further studies. Given the potential of both the framework and the interpretive synthesis approach in understanding and explaining any phenomenon of influence by synthesizing evidence from diverse literature, often from different disciplines and beyond maternal health, we urge further studies to explore their applicability.

## Appendix 1

**Table 6:** Search strategy

Search#	Search terms	Where (keywords)	Total hits	Date searched
PubMed				
1	((maternal health[Title/Abstract]) AND India[Title/Abstract]))	title/abstract	373	7 December 2016
2	(((accountability[Title/Abstract]) AND maternal health[Title/Abstract]) AND India[Title/Abstract])))	title/abstract	6	7 December 2016
3	(((maternal health[Title/Abstract] AND health system[Title/Abstract]) OR accountability[Title/Abstract]) OR governance[Title/Abstract]) AND India[Title/Abstract]	title/abstract	221	28 May 2017
ScienceDirect				
4	“accountability” AND “maternal health”	n.a.	52	7 December 2016
5	“maternal health” AND “India”	n.a.	131	7 December 2016
6	“maternal health” AND “health system” OR “governance” OR “accountability” AND “India”	n.a.	58	27 May 2017
7	Reference check	title/abstract	9	8 December 2016 29 May 2017

*n.a.* not applicable

## Appendix 2

**Table 7:** List of studies included

Author(s)	Title	Study objective/focus	Study year	Study area(s)	Source(s)
Bhattacharyya et al., 2015	‘Neither we are satisfied nor they’- users and provider’s perspective: a qualitative study of maternity care in secondary level public health facilities, Uttar Pradesh, India.	Perception of care on provision (factors) of quality maternity care in public health facilities	2014	Uttar Pradesh	Reference check
Chaturvedi et al., 2015	Does the Janani Suraksha Yojana cash transfer programme to promote facility births in India ensure skilled birth attendance? A qualitative study of intrapartum care in Madhya Pradesh.	Factors influencing skilled attendance at birth	2012	Madhya Pradesh—3 districts	Reference check
Dikid et al., 2013	Maternal and perinatal death inquiry and response project implementation review in India.	Implementation of MDR, including overview of delays and causes of maternal deaths	2009	Bihar, Rajasthan, and Odisha	PubMed
George A, 2007	Persistence of high maternal mortality in Koppal District, Karnataka, India: observed service delivery constraints.	Factors contributing to maternal deaths	2004	Karnataka—Koppal district	ScienceDirect
Human Rights Watch (HRW), 2009	No tally of the anguish: accountability in maternal health care in India.	Factors influencing maternal health-care utilization	2008–2009	Uttar Pradesh	Reference check
Iyengar et al., 2009	Pregnancy related deaths in rural Rajasthan, India: exploring causes, context, and care-seeking through verbal autopsy.	Factors contributing to maternal deaths	2002–2003	Rajasthan—a block	Reference check
Jat et al., 2015	Socio-cultural and service delivery dimensions of maternal mortality in rural central India: a qualitative exploration using a human rights lens.	Factors contributing to maternal deaths	2011	Madhya Pradesh—Khargone district	PubMed
Jeffery P & Jeffery R, 2010	Only when the boat has started sinking: a maternal death in rural north India.	Factors affecting maternal health	2002–2005	Uttar Pradesh—Muslim village in rural Bijnor district	PubMed and ScienceDirect
Jihesh V & Sundari Ravindran TK, 2015	Social and health system factors contributing to maternal deaths in a less developed district of Kerala, India.	Factors contributing to maternal deaths	2010–2011	Kerala—Wayanad district	ScienceDirect
Jose et al., 2014	Utilization of maternal health-care services by tribal women in Kerala.	Factors affecting utilization of maternal health-care services	2009–2010	Kerala—Wayanad district	PubMed
Kaur J, 2012	The role of litigation in ensuring women’s reproductive rights: an analysis of the Shanti Devi judgement in India.	Factors contributing to maternal death; gaps in health system	2010	Haryana	PubMed and ScienceDirect
Khan N & Pradhan MR, 2013	Identifying factors associated with maternal deaths in Jharkhand, India: a verbal autopsy study.	Factors contributing to maternal deaths	2006–2007	Jharkhand—5 districts	Reference check
Mahapatro M, 2015	Equity in utilization of health care services: perspective of pregnant women in southern Odisha, India.	Factors affecting utilization of maternal health-care services	2011–2012	Odisha—Gajam district	PubMed
Raj et al., 2015	Emergency referral transport for maternal complication: lessons from the community based maternal death audits in Unnao district, Uttar Pradesh, India.	Factors contributing to maternal deaths	2009–2010	Uttar Pradesh—Unnao district	Reference check
Sanneving et al., 2013	Health system capacity: maternal health policy implementation in the state of Gujarat, India.	Process of implementing maternal health policies at sub-national level	2009–2010	Gujarat	Reference check
Singh et al., 2015	Community based maternal death review: lessons learned from ten districts in Andhra Pradesh, India.	Implementation of MDR, including overview of delays and causes of maternal deaths	2011	Andhra Pradesh—10 districts	PubMed
Smith, SL, 2014	Political contexts and maternal health policy: insights from a comparison of south Indian states.	Sources of variation in maternal health policy and implementation at sub-national level	2006–2007	Tamil Nadu and Karnataka	ScienceDirect
Subha Sri B & Khanna R, 2014	Dead women talking: a civil society report on maternal deaths in India.	Factors contributing to maternal deaths	2012–2013	10 states—Assam, Jharkhand, Rajasthan, Gujarat, Odisha, West Bengal, Uttar Pradesh, Maharashtra, Bihar, Chhattisgarh	Reference check
Subha Sri et al., 2012	An investigation of maternal deaths following public protests in a tribal district of Madhya Pradesh, central India.	Factors contributing to maternal deaths	2010	Madhya Pradesh—Barwani district	ScienceDirect
Sudhinaraset et al., 2016	Decision-making for delivery location and quality of care among slum-dwellers: a qualitative study in Uttar Pradesh, India.	Factors influencing women’s decision to seek maternal health and quality of care received	2014	Uttar Pradesh—urban slums of Lucknow	Reference check
Vellakkal et al., 2017	A qualitative study of factors impacting accessing of institutional delivery care in the context of India’s cash incentive program.	Factors influencing access to JSY and institutional delivery	2013	Jharkhand, Madhya Pradesh, and Uttar Pradesh	ScienceDirect

## Abbreviations

ANC: Antenatal care
ANM: Auxiliary nurse-midwife
ASHA: Accredited Social Health Activists
JSY: Janani Suraksha Yojana
MDR: Maternal death review
MMR: Maternal mortality ratio
NRHM: National Rural Health Mission
PHC: Primary health center
PNC: Postnatal care
VHSNC: Village Health, Sanitation and Nutrition Committee
